# Classmate characteristics, class composition and children’s perceived classroom climate

**DOI:** 10.1007/s10389-017-0809-0

**Published:** 2017-05-20

**Authors:** Louise Persson, Mikael Svensson

**Affiliations:** 1 Centre for Research on Child and Adolescent Mental Health, Karlstad University, SE-651 88 Karlstad, Sweden; 2 Public Health Sciences, Karlstad University, Karlstad, Sweden; 3 Department of Health Metrics, Gothenburg University, Gothenburg, Sweden

**Keywords:** Children, Class composition, Classroom climate, Public health sciences, School health promotion

## Abstract

**Aim:**

A beneficial classroom climate is vital for school achievements, health, well-being, and school satisfaction. However, there is little knowledge as to how the classmate characteristics and class composition are related to the level of a perceived messy and disorderly classroom climate and whether the estimated relationships vary between different groups of children. The aim of the study was to explore the relationship between classmate characteristics as well as class composition and children’s perceived classroom climate.

**Method:**

Data from a cross-sectional survey administrated in 71 classes including 1,247 children in a mid-sized Swedish city were used. The analyses were conducted using multilevel models.

**Results:**

A class with a higher proportion of girls was associated with a lower likelihood of perceiving the classroom climate as messy and disorderly. Moreover, a higher proportion of immigrant children in a class was associated with a perception of a messier and disorderly classroom climate among non-immigrant children, but not among immigrant children themselves.

**Conclusion:**

Classmate characteristics and class composition deserve more research attention and can be important considerations when aiming to improve the classroom climate and children’s well-being in general.

## Introduction

In school, the classroom is where children spend most of their time, and a beneficial classroom climate is of substantial importance in establishing a supportive and healthy school environment (WHO 1991; Persson and Haraldsson 2013). It has been shown that an important aspect of the classroom climate is children’s own perception of it (Gillen et al. 2011; Hagquist 2012; Veerman 2015). It is, however, difficult to identify a universally agreed-upon definition of what is meant by “classroom climate” in the literature or to what a perception of a messy and disorderly classroom specifically refers (Duun and Harris 1998; Gillen et al. 2011). In this study, for example, we focus the question of children’s perceived messy and disorderly classroom climates as done before by Veerman. A poorer classroom climate may be a mix between work behavior (whether or not the children are able to listen to instructions and/or whether they can work properly in the classroom) and children’s disruptive behavior (Veerman 2015). A poorer classroom climate is seen as a serious threat to the learning environment (Veerman 2015), children’s school achievements, health, well-being (Holen et al. 2012), and school satisfaction (Persson et al. 2016). Therefore, a beneficial classroom climate is increasingly recognized in the policy world.

The classroom climate can encompass both the learning environment and the social relations in the classroom (Allodi 2010). Also, we know that positive or negative sets of behaviors, expectations, ambitions, and resources are generated among the children in the class, depending on how well classmates bond with one another (Östberg and Modin 2007). The school class is defined as having distinct boundaries, which indicates that the class members are well defined, and the size and composition of a class are *not* chosen by the children themselves (Butts 2008). Children’s background in the school class (such as socioeconomic status, resources, and attitudes to learning, etc.) also depends on the socioeconomic and cultural characteristics of each school area (Almquist 2011).

One country with a worrying trend regarding the classroom climate is Sweden (Skolverket 2012; OECD 2015), and the classroom discipline in Sweden is poor according to international comparisons (OECD 2015). A national investigation of Swedish children’s attitudes to school shows that approximately 60% of the Swedish children, aged 10 to 13, report that a messy and disorderly classroom climate is “sometimes” a problem, and only every fourth child reports that there are almost always or always low levels of a messy and disorderly classroom climate (Skolverket 2013). In a recent qualitative study, Swedish children also stated the desire for a more orderly classroom climate and improved classroom discipline (Persson et al. 2016).

However, even though we know that a beneficial classroom climate is vital for a whole range of issues (the learning environment, school achievements, well-being, and health) and that a child’s background is important for how the classroom climate is perceived, there is little knowledge as to how the classmate characteristics and class composition are related to the level of children’s perceived classroom climate. Some of the relatively scarce research in this field has shown that a larger proportion of girls in a class improves the school achievements of both girls and boys, decreases violence-related problems, and improves social relations within the classroom (Lavy and Schlosser 2011). In recent years, Sweden has seen, in relative per capita terms, one of the largest numbers of immigrants from distant countries and cultures. The demands on the Swedish school system to introduce, integrate, and educate immigrants have noticeably increased. However, hardly every second teacher thinks that he or she can meet these demands (Skolverket 2013). Often, the situation has also led to an increased need for resources for schools in areas with a high proportion of immigrants, along with increased demands on the teachers as educators (Bunar and Kallstenius 2007). A study by Veerman (2015) shows significant results when it comes to children’s perceived disruptive classroom climate and ethnic diversity in schools. Also, one of the four classroom variables measured related to classroom climate was, similar to this study, children’s perceived “noise and disorder” in the classroom.

In the literature exploring the role of sex composition and proportion of immigrants and the classroom climate, we have not been able to identify previous research on the heterogeneity of these factors, e.g., do boys and girls similarly report a poorer classroom climate in a class with a high proportion of girls or is this relationship confined to one of the sexes? The same is true for the proportion of immigrants in a class, i.e., does the relationship between this proportion and a perceived poorer classroom climate look similar between immigrants and non-immigrants? Altogether, classmates’ characteristics and the composition of the school class need to be further investigated, especially with regard to the potential heterogeneity in the relationships between classmate characteristics and children’s perceived classroom climate.

The aim of the study is therefore to contribute to the issue at hand by exploring the relationship between classmate characteristics as well as class composition and children’s perceived classroom climate. This is done by analyzing: (1) how the individual-level variables for grade, sex, immigrant status, and single parent households are associated with children’s perceived classroom climate, 2) how the class composition, measured as the proportion of girls, the proportion of immigrants, and the proportion of single-parent children, is associated and interacts with the individual child’s perception of the classroom climate, and (3) whether the estimated relationships vary between different groups of children, i.e., potential heterogeneity.

## Method

### Data

The data collection was carried out at one time point in May 2011 in a middle-sized municipality in central Sweden. A total of 1247 children, aged 10 to 12 years, in grades 4–5, in 71 schoolclasses, from all 21 elementary schools in the municipality participated. The response rate was 84%. Due to the young age of the participants, a signed informed consent from all the parents was required for the children to participate in the study. All participants received both written and oral information about the aim of the study, the design, the voluntary nature of participation, the right to stop their participation at any time, and the confidential treatment of data. The Ethics Committee at Uppsala University in Sweden reviewed the study, and no objections were raised (no. C2009/623).

### Procedure

The questionnaire was distributed to the children in a classroom setting by research members during regular class hours. The questionnaire took about 60 min to complete and included questions about health, school climate, and social relationships. All answers were anonymous, and the children had the possibility to withdraw at any time.

### Outcome and explanatory variables

#### Outcome variable

##### Classroom climate

To begin with, instead of using reports from school staff we measured classroom climate using the children’s reports. The outcome variable of interest is based on one variable where the children were asked: “Do you think it is messy and disorderly in the classroom?” (Hagquist 2012). The categories were “mostly,” “sometimes,” “seldom,” and “never.” Due to the young age of the children the questionnaire could only contain a small number of variables, and one variable about the classroom climate was included. We used this variable to compose a binary dummy outcome variable *Messy and disorderly classroom climate*, which takes the value of zero if the child said that it was “seldom” or “never” messy and disorderly in the classroom and the value of one if the child responded that it was “sometimes” or “mostly” messy and disorderly in the classroom. As shown in Table 1, 60% of the children perceive the classroom climate to be messy and disorderly.^1^

**Table 1:** Summary statistics

Variable name	Description	Proportions
Outcome variable		
Messy and disorderly classroom climate	=1 if mostly or sometimes a messy and disorderly classroom climate	0.60
Individual-level explanatory variables		
Grade 5	=1 if pupil in grade 5, 0 if pupil in grade 4	0.49
Female	=1 if pupil is a girl	0.49
Immigrant	=1 if pupil is first or second generation immigrant	0.19
Single parent	=1 if pupil lives in a single-parent household	0.16
Class-level explanatory variables		
High female proportion	=1 if the proportion of girls among classmates >60%	0.17
Low female proportion	=1 if the proportion of girls among classmates <40%	0.19
Immigrant proportion	Proportion of immigrants among classmates	0.19
Single parent proportion	Proportion of single parent households among classmates	0.16

#### Explanatory variables

##### Individual-level explanatory variables

A few of the variables in the questionnaire referred to children’s background, which we use as a basis for analyzing how factors at the individual level and group levels may affect the perception of a messy and disorderly classroom climate: grade/school year, sex, immigrant status, and single parent household. In Table 1, we see that 49% of the sample is from grade 5 (51% from grade 4) and that 49% are girls. Further, 19% of the children are defined as immigrants based on three different variables regarding country of birth (“In which country were you born?” “In which country was your mother born?” and “In which country was your father born?”). We defined an immigrant as a first- or second-generation immigrant if the child was born in another country or if at least one of the parents was born in another country (independent of which ethnic group children are identified with). Finally, 16% of the children live in a single parent household, which derives from the variable concerning whether or not the children lived with both of their parents, had equal time with their mother and father (for example, alternating weeks), or were with only one of their parents most of the time. We define a single parent household as one in which the child lives with only one of their parents most of the time (predominantly their mother).^2^

##### Class-level explanatory variables

We coded the class-level variables as the average response to each individual-level variable among a child’s classmates. For example, for a class of 20 children (numbered 1 to 20) the proportion of immigrants in this class for child 1 is based on the proportion of immigrants among child 2 to 20. For child 2, we took the proportion of immigrants among child 1 and 3–20, etc. For the proportion of girls, we instead created two different dummy variables, a *high female proportion* and *low female proportion*, since our analyses showed that models treating the proportion of girls as non-linear were preferred compared to models treating the proportion of girls in a linear fashion, i.e., with regard to the statistical fit.^3^ For *immigrant proportion* and *single parent proportion*, the mean response is the same as for the individual-level variables but the standard error is smaller.

### Empirical strategy

We used multilevel models for dichotomous/binary responses. Considering the hierarchical structure of the data, children grouped into classes and schools, modeling this at the individual level may violate the assumption of independence of observations. The potential problem is typically that it can lead to deflated standard errors (i.e., overstate the statistical significance of coefficient estimates). To address this concern, we used a random intercept multilevel logit model where children are nested into classes. We estimated fixed effects at both the individual level (e.g., the sex of the child) and the class level (e.g., the proportion of girls among classmates); at the same time we estimated a random effect at the class level, and we allowed the intercept to vary across classes. The random effect can be interpreted as the sum of the combined effects of omitted child-specific covariates correlated within classes causing some children to perceive the classroom climate to be more or less messy and disorderly. The benefits of the multilevel approach include that we can control for dependencies in the data that are the result of the fact that children in the same class share the same classroom environment (affecting our relationships of interest), and we were also able to include fixed effects at both the individual and class level (Rabe-Hesketh and Skrondal 2008).^4^ The estimations in this study are the result of using the binary response multilevel model routine in Stata v.13.

## Results

Table 2 shows the results for three different multilevel logit models. In model (a), only individual-level variables for grade, sex, immigrant status, and single parent households are included. In model (b), only classroom variables measuring the proportion of girls, the proportion of immigrants, and the proportion of single parent households are included, whereas in model (c) both the individual-level and classroom variables are included. We conducted the separate regression to explore the robustness of the estimated coefficients across different model specifications.

**Table 2:** Multilevel regression results for the outcome variable “Messy and disorderly classroom climate”

Variable	Odds ratio (95% CI)		
	Model (a)	Model (b)	Model (c)
Grade 5	1.25 (0.76–2.05)	-	1.27 (0.77–0.207)
Female	1.95^**^ (1.50–2.56)	-	1.94^**^ (1.47–2.55)
Immigrant	1.08 (0.75–1.54)	-	1.07 (0.75–1.54)
Single parent	1.08 (0.74–1.55)	-	1.12 (0.76–1.65)
High female proportion >60%	-	0.42^**^ (0.23–0.78)	0.47^*^ (0.25–0.88)
Low female proportion <40%	-	0.90 (0.52–1.56)	0.71 (0.40–1.27)
Immigrant proportion	-	0.47 (0.10–2.29)	2.37 (0.47–11.88)
Single parent proportion	-	0.38 (0.04–4.00)	2.36 (0.19–28.90)
Constant	1.08 (0.72–1.60)	1.46 (0.89–2.40)	0.94 (0.53–1.67)
Random-intercept estimate	1.09^**^ (0.86–1.37)	1.03^**^ (0.81–1.31)	1.05^**^ (0.83–1.34)
Log likelihood	−742.91	−751.11	−738.68
Intraclass correlation	0.27^**^	0.24^**^	0.25^**^

**Note:** ^***^ *p* < 0.001, ^**^ *p* < 0.01, ^*^ *p* < 0.05. The model is based on 1232 children divided into 71 classes

The random intercept parameter is statistically significant in all models, indicating that there is substantial between-class variation that the model captures and, i.e., a multilevel approach is suitable. The estimates of the intraclass correlation (ICC), which is a measure of the correlatedness within classes, are 0.27, 0.24, and 0.25, respectively. The ICC in the null model (not shown in the table), i.e., only estimating the random intercept, is 0.25 and thus indicates that there is a correlation within classes.

In model (a), we see that at the individual level being a girl is statistically significantly related to a higher likelihood of perceiving the classroom climate as being characterized as messy and disorderly with an odds ratio (OR) of 1.95. Other individual-level variables are not statistically significantly related to the outcome variable. In model (b), we see that children attending a class where the proportion of female classmates is high are more likely to consider characterizing the classroom climate as less messy and disorderly (OR: 0.42). Hence, controlling for class-level random effects and the other class-level variables, it is clear that attending a class with a higher proportion of girls is associated with perceiving a less messy and disorderly classroom climate. Finally, in model (c), the results show that both effects that were statistically significant in model (a) and (b) are statistically significant in a model controlling for both individual- and class-level variables. Regarding the class-level variables, the only statistically significant effect is still that attending a class with a high proportion of girls is related to perceiving the classroom climate as less messy and disorderly.

The odds ratios are difficult to interpret regarding the magnitude of the effects, and we therefore focus on estimates of predicted probabilities. In a post-estimation based on model (c), including both the estimated fixed effects as well as the random school-level effect, it can be shown that the predicted probability that a child will perceive the classroom climate to be messy and disorderly is almost 50% if the child attends a class with a female proportion of >60%. On the other hand, if a child attends a class where the proportion of girls is below 60%, the predicted probability that the child will perceive the classroom climate as messy and disorderly is on average 70%.

Table 3 shows the regression results of the analyses, including interaction effects between the individual and the respective class-level variable. To be more specific, it shows the estimation results from model (c) of the regressions conducted, including interactions between being a girl and a high and low female proportion, being an immigrant and the proportion of immigrants, and living in a single parent household and the proportionof single parent households. Models (c1) to (c3) include the different interaction effects independently, while model (c4) includes all three interaction effects in a joint model.

**Table 3:** Regression results with interaction effects between individual- and class-level variables

Variable	Odds ratio			
	Model (c1)	Model (c2)	Model (c3)	Model (c4)
Grade 5	1.28	1.31	1.28	1.32
Female	2.10^***^	1.95^***^	1.95^***^	2.10^***^
Immigrant	1.07	2.25^***^	1.07	2.20^***^
Single parent	1.12	1.07	1.59	1.47
High female proportion >60%	0.53^*^	0.48^**^	0.47^**^	0.53^*^
Low female proportion <40%	0.78	0.73	0.70	0.78
Immigrant proportion	2.33	11.22^**^	2.55	11.34^**^
Single parent proportion	2.44	3.42	3.36	4.70
Girl × high female proportion	0.78	-	-	0.79
Girl × low female proportion	0.82	-	-	0.85
Immigrant × immigrant proportion	-	0.04^***^	-	0.05^***^
Single parent × single parent proportion	-	-	0.14	0.18
Constant	0.90	0.68	0.87	0.62
Random-intercept estimate	1.06^***^	1.08^***^	1.05^***^	1.08^***^
Log likelihood	−738.43	−733.49	−737.80	−732.64

**Note:** ^****^ *p* < 0.001, ^***^ *p* < 0.01, ^**^ *p* < 0.05, ^*^ *p* < 0.01 The model is based on 1232 students divided into 71 classes

**Fig. 1:**
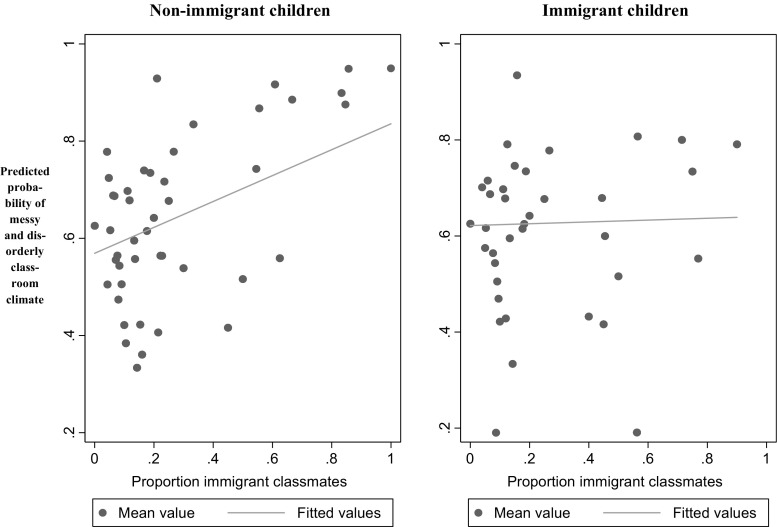
Predicted probability of a messy and disorderly classroom climate according to the proportion of immigrant classmates

The results in Table 3 reveal two additional findings compared to Table 2. The relationship that the children perceive classrooms with a higher proportion of girls to be less messy and disorderly holds for both boys and girls, i.e., there is no difference between the sexes in this result. The second noteworthy result shown in Table 3 is the statistical significances and, in terms of the statistical relevance and magnitude, a large interaction effect between being an immigrant and the proportion of immigrants among classmates. Interpreting the main effect of *Immigrant proportion* in model (c2) and (c4), we see that children who attend a class with a higher proportion of immigrant classmates are more likely to consider the classroom climate to be messy and disorderly. However, this effect completely disappears (and is almost reversed) if we correlate the proportion of immigrants with whether or not the child is an immigrant him- or herself. Thus, the majority of non-immigrant children attending a class with more immigrant children consider such a class to be more messy and disorderly, whereas immigrant children’s perception of a messy and disorderly climate is unrelated to the proportion of classmates being immigrants. To demonstrate these results more clearly, we estimate the predicted probability of the child having a *Messy and disorderly classroom climate = 1* (based on model c4) for non-immigrant and immigrant children, respectively. The results are shown in Fig. 1 below. As we can see, the likelihood of perceiving the classroom climate to be characterized as messy and disorderly increases for non-immigrant children with higher proportions of immigrant classmates. Using a rough approximation, for every ten percentage point increase in the proportion of immigrant classmates, the probability that a non-immigrant child will perceive the classroom to be messy and disorderly is three percentage points higher.

## Discussion

The aim of the study was to explore the relationship between classmate characteristics as well as class composition and children’s perceived messy and disorderly classroom climate. From an educational perspective, as well as from a public health perspective, a messy and disorderly classroom climate can have substantial negative consequences with regard to children’s learning environment, educational achievements, well-being, and health (Currie et al. 2008; Gustafsson et al. 2010).

We found that girls tend to have a higher likelihood of perceiving the classroom climate to be characterized as messy and disorderly. Whether a boy or a girl, we found that a higher proportion of female classmates is associated with a perception of a classroom climate that is less messy and disorderly. This is in line with previous research that showed that a class with a higher proportion of girls is associated with a lower level of messy and disorderly classroom climates and violence, better within-class social relationships, and better child-teacher relationships (Lavy and Schlosser 2011). The differences between girls and boys in perceiving the classroom climate as messy and disorderly may depend on differences and inequalities between the sexes in the Swedish schools (SOU 2010:99; SOU 2014:6). There are, for example, noted differences in school achievement, preferences, and experiences between girls and boys in school. Boys perform less well than girls (Skolverket 2012) and are more involved in fights, victimizations, and homophobic harassments (HBSC 2014; SOU 2014:6), and ADHD diagnoses are more common among boys (Rucklidge 2010). Consequently, boys can be seen as messier and more trouble prone than girls in the classroom. Another explanation for the result could be that boys simply put less effort into schoolwork and are more unfocused, which has been deliberated in the literature (SOU 2014:6). For example, Phoenix (2003) describes a concern related to boys’ vague attitudes toward schoolwork in the form of ‘anti-studying cultures.’ Anti-studying cultures describe how boys in particular can seek status and popularity by investing in a form of masculinity in which it is essential to show toughness and distance to what is perceived as being an “academic high achiever.” It has also been shown that it can be beneficial for a high-achieving child’s well-being to be in a class with high-achieving homogeneous classmates, while low-achieving children can benefit more from being in heterogeneous classes (Belfi et al. 2012). In sum, this knowledge deserves to be better investigated in the future, for example, through interviews with children and teachers, to obtain their views on possible explanations of the issue.

Further, we found that children’s perceived messy and disorderly classroom climates are related to the proportion of immigrants in a class. However, this was only statistically significant if taking heterogeneity into account. Non-immigrant children attending a class with a higher proportion of immigrant classmates were more likely to consider the classroom climate to be messy and disorderly. For immigrant children there was no relationship between the proportion of immigrant classmates and perceived messy and disorderly classroom climates. This is partly in line with previous research, e.g., Arum et al. (2012), which found that a higher proportion of immigrant children is related to disciplinary problems in the classroom. Likewise, a recent study demonstrated that ethnic diversity is significantly associated with a messy and disorderly classroom climate (Veerman 2015). Potential reasons may include language differences, which may be perceived as worse for a non-immigrant child, if attending a class with more immigrant children, than for an immigrant child, as elaborated in a study by Diette and Uwaifo Oyelere (2014). Immigrant children might also have experienced a range of traumatic events (i.e., assaults, serious accidents, abuse), which can have a negative impact on their functioning in the classroom (Kataoka et al. 2012) and may result in a class with more immigrant children being perceived as messier and more disorderly if children are not immigrants themselves.

The results of this study could be important to consider when planning teaching, for example, when implementing school activities aiming to strengthen the social relations between children in school, when combating anti-studying cultures, and when working toward a more inclusive classroom climate. In short, paying attention to the classmate characteristics and class composition might be an effective measure for improving the classroom climate and be important for children’s learning environments, school achievements, school satisfaction, well-being, and health.

## Limitations

All children in grades 4–5 (aged 10 to 12 years) in a municipality were asked to participate, which means that the results include all middle school children that school year, which may facilitate potential generalizations (Djurfeldt et al. 2009). The questionnaire was also pre-tested among grade 4 children (aged 10 to 11), and the schools were revisited in order to include absentees from the regular data collection session. Further, how the children perceived the variables was checked by writing down the queries the children had about the variables, i.e., which variables they did not understand and why they did not understand them. Later, of importance for the study validity, all responses from the children in all classes were checked to identify problematic variables and try to define the potential problems. The most important limitation of the study, in terms of interpretations and conclusions, relates to the fact that it is cross-sectional, and we did not try to infer causal estimates using some form of quasi-experimental approach. This implies that the results need to be interpreted as descriptive (“risk factors”), i.e., it shows in what type of classes we may expect more (or less) perceived messy and disorderly classroom climates, but it is not necessarily causally related to our individual- or class-level variables. An additional potential limitation concerns the dichotomization of the outcome variable. Putting the categories “mostly” and “sometimes” together may lead to an “enlarged” problem, but it is difficult to determine what an “acceptable” level of messy and disorderly classroom climate is. Moreover, a possible weakness might be that ‘classroom climate’ can have many meanings depending on who is asked (for example, teachers or children), but it also is argued that a specific definition of the concept may be a limiting factor when it comes to research (Duun and Harris 1998). Another limitation may be that the classroom outcome measure only contained one variable: “Do you think it is messy and disorderly in the classroom?” It would have been preferable if the questionnaire had included more specific questions about the classroom climate. Overall, the concept of the classroom climate needs to be better investigated and followed up in future research, preferably through both quantitative and qualitative interviews with children, asking for their view of the question of interest and what might be included in a messy and disorderly climate. This would also help to interpret the results from this study.

## Conclusions

From a public health, school health promotion, and educational perspective, it is essential that the school setting functions as a supportive environment for academic achievement, well-being, and health. This study explored the relationship between children’s perceived messy and disorderly classroom climate and classmate characteristics, class composition, and cross-level interactions between them. It shows that the proportion of boys and the proportion of immigrants in a class are significant risk factors for a messier and more disorderly classroom climate. First, a school class with a higher proportion of boys is more likely to be associated with a higher likelihood of perceiving the classroom climate as messy and disorderly. Second, a school class with a higher proportion of immigrant children is associated with a messier and more disorderly classroom as perceived by non-immigrant children, but not among immigrant children.

## Footnotes

1 In the final analyses, we dichotomized the outcome variable mainly because it simplifies the interpretation of the results. However, we also conducted analyses in the study where we used a different threshold for the dichotomization of the outcome variable as well as ordinal regressions (results available upon request), all with qualitatively similar results as reported in the study.

2 We also conducted analyses in this study in which we distinguished between children living with both of their parents or with both their mother and father but on alternating weeks and those living only with one parent. However, the results indicate that there were no significant differences between children living with both parents and children living with their separated mothers and fathers on alternating weeks. Hence, these two family structures are conflated in the analyses reported in the study.

3 The proportion of girls in a class does not vary to any significant extent beyond a 25% to 75% interval.

4 Given the data in this study, it would also be possible to consider a three-level mixed model where each child is clustered within classes and within schools. However, here, our analyses showed that there was no significant benefit in introducing the school-level effect once the class-level had been introduced. Hence, we stayed with the two-level model where each child is clustered within classes.
